# Pipeline for Large-Scale Microdroplet Bisulfite PCR-Based Sequencing Allows the Tracking of Hepitype Evolution in Tumors

**DOI:** 10.1371/journal.pone.0021332

**Published:** 2011-07-05

**Authors:** Alexander Herrmann, Andrea Haake, Ole Ammerpohl, Idoia Martin-Guerrero, Karol Szafranski, Kathryn Stemshorn, Michael Nothnagel, Steve K. Kotsopoulos, Julia Richter, Jason Warner, Jeff Olson, Darren R. Link, Stefan Schreiber, Michael Krawczak, Matthias Platzer, Peter Nürnberg, Reiner Siebert, Jochen Hampe

**Affiliations:** 1 Institute of Internal Medicine I, Christian-Albrechts University, Kiel, Germany; 2 Institute of Human Genetics, Christian-Albrechts University, Kiel, Germany; 3 Genome Analysis Group, Fritz-Lipman Institute for Ageing Research, Jena, Germany; 4 Cologne Center for Genomics, University of Cologne, Cologne, Germany; 5 Institute of Medical Informatics and Statistics, Christian-Albrechts University, Kiel, Germany; 6 RainDance Technologies, Lexington, Massachusetts, United States of America; The Chinese University of Hong Kong, Hong Kong

## Abstract

Cytosine methylation provides an epigenetic level of cellular plasticity that is important for development, differentiation and cancerogenesis. We adopted microdroplet PCR to bisulfite treated target DNA in combination with second generation sequencing to simultaneously assess DNA sequence and methylation. We show measurement of methylation status in a wide range of target sequences (total 34 kb) with an average coverage of 95% (median 100%) and good correlation to the opposite strand (rho = 0.96) and to pyrosequencing (rho = 0.87). Data from lymphoma and colorectal cancer samples for *SNRPN* (imprinted gene), *FGF6* (demethylated in the cancer samples) and *HS3ST2* (methylated in the cancer samples) serve as a proof of principle showing the integration of SNP data and phased DNA-methylation information into “hepitypes” and thus the analysis of DNA methylation phylogeny in the somatic evolution of cancer.

## Introduction

Epigenetic mechanisms are key to the control of local transcriptional activity [1]. Methylation of cytosine residues at the carbon 5 position (5^me^C) in the context of CpG motifs is one of the best studied epigenetic marks. DNA methylation in mammalian genomes can affect different genomic areas, such as repeat regions, gene promoters and the gene body. Whereas methylation of DNA repeats is assumed to regulate genome stability, DNA methylation of CpG islands in gene promoters and the gene proper have been inversely linked to gene expression [2], [3].

Alterations in DNA methylation are not only crucial for normal development but are also characteristic of several physiologic and disease associated mechanisms, including ageing, imprinting defects and genomic instability syndromes [4]. Altered DNA methylation patterns have been particularly well studied in cancer [5], [6], [7]. Virtually all cancers are in some way associated with aberrant DNA methylation. High levels of DNA methylation at gene promoter regions have been described in hematologic neoplasms like germinal-center B-cell derived lymphomas and in solid tumors like colorectal, prostate and brain cancer [8], [9], [10]. Initially, altered methylation has been assumed to be one of the key silencing mechanisms for tumor-suppressor-like genes [11]. Nevertheless, increasing evidence suggests that in cancer DNA-methylation is a phenomenon encompassing a wide array of gene types [10], [12]. Remarkably, among methylation targets in various types of cancer, target genes of the polycomb repressive complexes (PRC1 and PRC2) in stem cells are strongly enriched. These genes regulate key developmental processes and play an important role in differentiation and the maintenance of cell fates [13], [14], [15]. Polycomb target gene methylation has been reported as a specific pattern of *de novo* methylation in cancer [16], [17], although the stability and mechanistic interaction of this gene set in evolving cancer cells remains to be clarified [18].

Whereas much is known about the somatic patterns of DNA methylation in cancer only scarce data exist that link genetic (e.g. DNA based) predisposition to cancer to the somatic manifestation of clonal outgrowth. Given that many predisposing loci are associated with a restricted spectrum of cancers, it is intriguing to speculate that epigenetic factors are involved in the somatic manifestation of disease predisposition. This concept recently leads to the definition of “hepitypes”, i.e. the description of (DNA-based) haplotypes which change the local epigenetic landscape [19], [20], [21]. The analysis of such hepitypes may provide an in-depth understanding of the biology of a given locus with a detailed analysis of both sequence variation and differential DNA methylation [3]. This is especially important in the context of the many large regions identified as polygenic risk factors for cancers such as colorectal, breast and prostate cancer through recent genome-wide association studies [22]. Ideally a parallel, deep analysis of somatic variation and methylation over continuous stretches of DNA should be conducted in order to understand the interaction of germline risk factors, somatic genetic and epigenetic evolution within the tumor [23], [24], not the least because altered methylation may affect cytosine residues outside a CpG context [25].

A number of methodologies for the analysis of methylation in the human genome are available including assays based on enzymatic digestion, affinity enrichment and sodium bisulfite enrichment, which have been recently reviewed [26], [27], [28]. Although a number of methods have been developed in order to increase throughput and ease of methylation analysis, no universally applicable technology has emerged as of yet. One possible approach uses bisulfite conversion; i.e. treatment of genomic DNA with sodium bisulfite (BS) to convert cytosine, but not methylcytosine, to uracil, and subsequent sequencing. Single-base methylation analysis was previously achieved using this method for proportions of the human genome [29], [30] and on a whole-genome level in *Arabidopsis thaliana* [31] and recently also in mammalian cells [25]. While these studies demonstrated the applicability of BS sequencing on the whole-genome level, some drawbacks including the cost of sequencing and bioinformatic alignment remain. Cost and coverage are particularly important if a quantitative assessment of the relative methylation of individual sites in a multiclonal tumor sample is attempted. We have thus adapted the recently described microdroplet PCR [32] to BS treated target DNA and established a pipeline for the simultaneous assessment of DNA sequence analysis and methylation and demonstrate the practical applicability using samples from colon cancer and follicular lymphomas.

## Methods

### Primer design pipeline for bisulfite-converted target DNA

Target regions for the methylation analysis with a total of 34083 base pairs of sequence were selected as detailed in Table S1. Forward and reverse strands of the target sequence were bisulfite converted *in silico*. The converted DNA was fragmented *in silico* into 200, 300 or 400 bp segments using a spacing of 5 bp. These segments were submitted to Primer3 [33] using the following parameters: target primer melting temperature 56±2°C, maximal difference between primer melting temperature 2°C, optimal primer size 20 bp [min = 18, max = 25], optimal GC content 60°C [min = 20°C, max = 90°C] and amplicon range search segment size ±50 bp. For each sequence segment, the five best primer pairs based on the Primer3 quality score were selected. In order to minimize allelic amplification bias, primers covering the location of a HapMap annotated SNP with a minor allele frequency >0.1 or a CpG island were removed. Redundant primer pairs were eliminated and the remaining primers checked by e-PCR [34] against the whole bisulfite converted genome. All primer pairs yielding off-target products of up to 2 kb were filtered out. Using a target coverage of 2-fold for the 300 bp design and a 3-fold coverage for the 200 bp and 400 bp amplicon design, primers were each selected by increasing GC-content to the desired coverage. For regions, where the above described criteria failed to produce the target coverage, the melting temperature criteria were relaxed to 56±6°C and the procedure repeated. A list of all primer pairs and their normalized sequencing yield is provided in Table S2, thus providing an indication of efficiency for each PCR and sequencing reaction.

### Design pipeline for non-converted DNA

Chromosomal coordinates (NCBI build 36/hg18) for the target regions were submitted to the RainDance Technologies primer design pipeline as described [32]. In brief, PCR primer pairs were designed for the target regions using a Primer3-based algorithm [33]. Known SNPs were masked prior to PCR primer design (dbSNP 129). PCR primers were designed to have a melting temperature (T_m_) of 58°C±1°C with an optimal length of 20 bases and an upper amplicon size of 600 base pairs. For target regions which were greater than 600 bp or for which a single amplicon of 600 bp was not able to be designed within parameters, an amplicon tiling strategy was used to cover the region. All PCR primer pairs were checked by *in silico* PCR [35] to identify regions of the genome other than the intended target region which could potentially be amplified. A total of 249 PCR primer pairs were designed that covered the entire 58716 bp of the target region and thus slightly more sequence than the converted design. All CpGs that are created through allelic variation in the samples are marked separately in Table S3. An overview of all variants detected in this study is provided in Table S4.

### Samples and bisulfite conversion

For the proof of principle experiments, DNA was prepared from tumor (CRC-TU) and normal tissue (CRC-NT) in a 65 year old male colon cancer patient. The cancer showed no evidence of microsatellite instability. In addition, we studied two lymph node specimens of a female patient initially diagnosed at age 60 years with follicular lymphoma (FL) stage IV with bone marrow involvement [36]. The specimens analyzed were diagnosed with typical FL1/2 obtained at relapse after partial remission achieved with anthracycline-containing chemotherapy one (FL-R1) and four (FL-R2) years after initial diagnosis. The FL hallmark translocation t(14;18)(q32;q21) was detected by FISH using the LSI IGH/BCL2 probe (Abbott) in 39% and 84% of nuclei in the both samples, respectively. Breakpoints affecting the BCL6 and MYC loci as well as IGH-MYC fusion were absent in both specimens by interphase FISH using commercially available probes (LSI BCL6 BAP, LSI MYC BAP, LSI IGH/MYC all of Abbott). Lymphoma cells expressed CD20, CD10, BCL2 and BCL6 in accordance with the diagnosis.Tissues samples were used in accordance with the protocols of the Colon Cancer Network and HämatoSys Networks for which IRB approval of the Ethics Committee of the Medical Faculty of the Christian-Albrechts-University Kiel was obtained. All sample DNAs were bisulfite converted using the EpiTect Bisulfite Kit (Qiagen, Hilden, Germany) according to the manufacturer's instructions. A verification of successful bisulfite conversion is shown in Figure S1.

### Droplet PCR and Optimization

**Figure 1:**
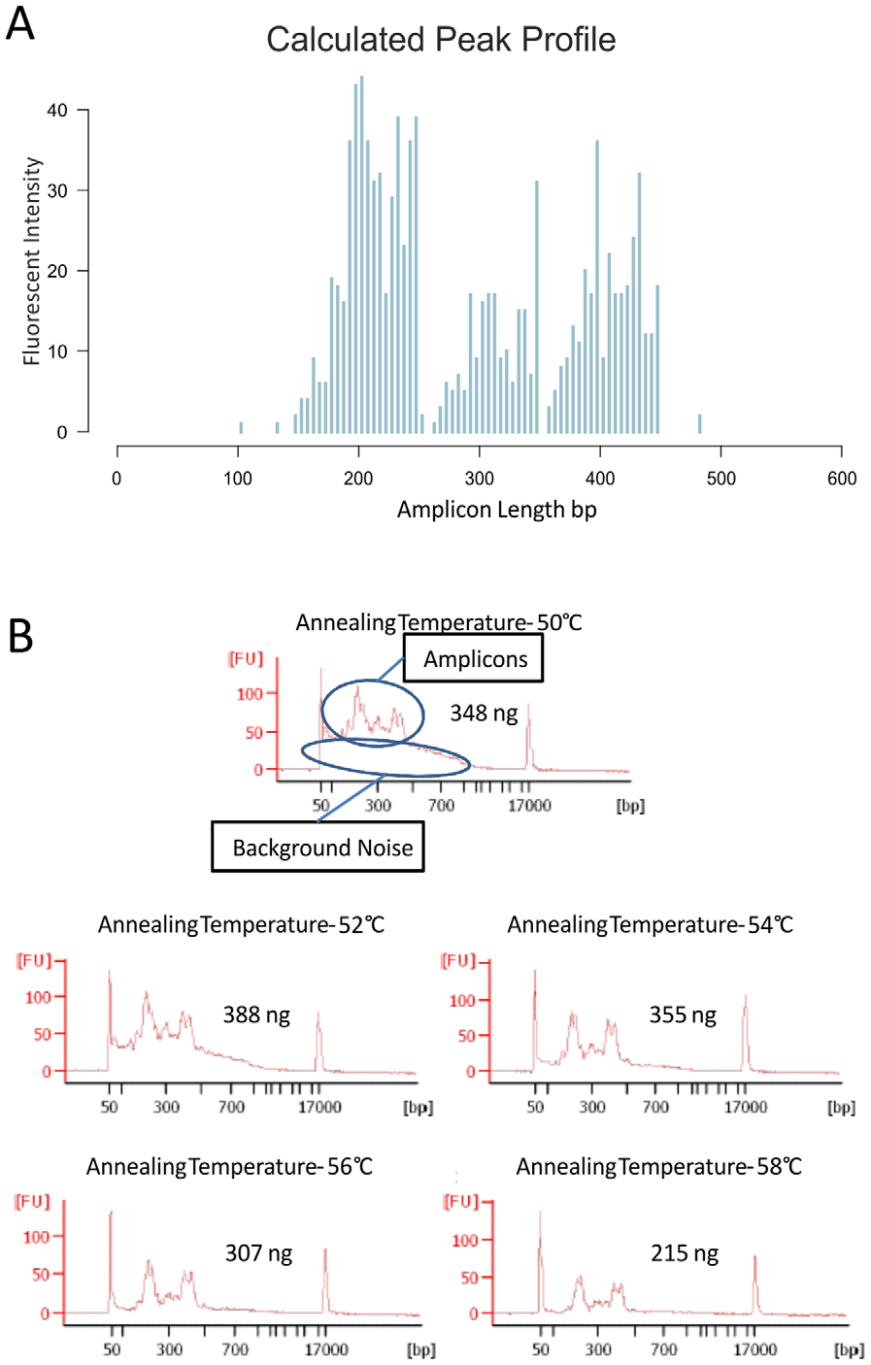
Annealing temperature optimization of the primer droplet library. Panel A – The calculated peak profile created by generating a histogram of the known amplicon sizes and numbers and assuming equal representation of all the amplicons after PCR amplification. Panel B – The Agilent Bioanalyzer traces of merged samples PCR amplified with different annealing temperatures. The amplicon peaks and background noise are highlighted for the 50°C annealing temperature. Background noise decreases until it disappears at 56°C. Increasing the annealing temperature to 58°C only results in a decrease in amplicon yield.

PCR primers designed to amplify the desired target regions from bisulfite treated DNA were synthesized, emulsified into a primer droplet library and merged with the target DNA of interest on the RDT 1000 instrument (RainDance Technologies, Lexington, MA/USA) as described [32]. In order to optimize both template buffer conditions and PCR cycling conditions, several sequence enrichment merges were performed varying both the MgSO_4_ concentrations and the annealing temperature. Following amplification and breaking of the emulsion, the sequence enriched amplicons were purified over a Qiagen MinElute column (Qiagen AG, Hilden, Germany) and eluted in 11 µL of EB buffer from the Qiagen kit. One µL of the eluted amplicons was run on an Agilent Bioanalyzer (Agilent, Santa Clara, Ca/USA). The optimal annealing temperature and MgSO_4_ concentration was determined by comparing the relative amplicon yields between samples at different annealing temperatures and MgSO_4_ concentrations and by the amount of background noise generated by comparing the actual Agilent Bioanalyzer profiles to the calculated peak profile. The calculated peak profile is a graphical representation of the expected shape of the Agilent Bioanalyzer peak profile created by generating a histogram from the known amplicon sizes and numbers (Figure 1, Panel A). The shape of the peak profile represents what the Agilent Bioanalyzer profile should look like if all amplicons designed are present at equimolar concentrations Figure 1 shows both the calculated peak profile (Panel A) and the actual Agilent Bioanalyzer traces for the merged samples run at different annealing temperatures (Panel B). At an annealing temperature of 50°C, the expected amplicon profile is present, however, there is a large amount of background noise present presumably due to off target amplification products at this lower annealing temperature. As the annealing temperature is increased, the background noise is decreased until it disappears at an annealing temperature of 56°C. Increasing the temperature to 58°C only decreases the amplicon yield from 307 ng to 215 ng without changing the background noise. Using this data an annealing temperature of 56°C was selected. The same procedure was used to determine the optimal MgSO_4_ concentration by keeping the annealing temperature constant at 56°C and varying the MgSO_4_ concentration from 1.8 mM to 2.5 mM (data not shown). Using this method it was determined that the optimal MgSO_4_ concentration for PCR amplification at an annealing temperature of 56°Cfor this primer droplet library was 2.5 mM.

### Library sequencing

Sequencing libraries were prepared according to Roche's rapid library preparation method manual (Roche, Hartford, CT/USA, release October 2009) with the following modifications: 134–288 ng of non-converted or bisulfite-converted micro droplet PCR products were used as starting material. The samples were directly subjected to fragment end repair (step 3.2) without prior nebulisation. Roche's MID-containing rapid-adapters were ligated to the samples. The fragment removal step (step 3.5) was replaced by purification with AmPure beads in order to remove excess adaptors but to retain the smaller RainDance products. The sample volume was adjusted to 50 µl with EB-Buffer (Qiagen) and the sample was added to 90 µl of AmPure XP beads (Agencourt Biosciences, Beverly, MA/USA). The sample was vortexed and incubated for 5 min at room temperature. The tube was placed for 3 min on a magnetic particle concentrator to pellet the beads. The supernatant was removed and the beads were washed two times with 500 µl 70% ethanol. Residual ethanol was removed by incubation at 37°C for 15 min. The purified sample was eluted with 50 µl TE-Buffer. Rapid libraries were quantified and diluted according to the rapid library protocol. Equal quantities of the non-bisulfite-converted samples were pooled. Titrations of the single bisulfite-converted libraries and the pooled non-bisulfite-converted libraries were performed according to Roche's emPCR method manual-Lib-L SV (October 2009). For the final bead enrichment (according to Roche's emPCR method manual-Lib-L LV, October 2009) a copy-per-bead ratio of 0.5 was used for the bisulfite-converted libraries and a copy-per-bead ratio of 1.5 was employed for the non-bisulfite-converted pool yielding enrichment rates between 7% and 12%. For each bisulfite-converted library and the pool of non-bisulfite-converted libraries a full sequencing run was performed according to Roche's protocol (Sequencing Method Manual, October 2009). Since reads from the non-bisulfite converted sample FL-R2 were underrepresented, sequencing of this library was repeated individually on 1/2 sequencing plate. Read data was extracted using the shotgun setting of the Roche analysis module.

### Analysis Pipeline

The data from the unconverted libraries were analysed as described previously [32]. The sequence reads from the converted libraries were compared to the total primer library using the string-search algorithm suggested by Ukkonen [37] allowing for a maximum of two differences per primer. Sequences without matches in the primer library, accounting for approximately 5% of reads, were eliminated. Subsequently, each sequence read was aligned to all potential amplicons compatible with the primer sequences using a modified dynamic programming alignment algorithm [38]. All sequence reads yielding a minimum of 50 matching bases and greater than 95% sequence identity to a target amplicon were selected for further analysis. This criterion was chosen in accordance to previous publications [38] to allow for variation (i.e. the 95% identity criterion to allow for differential methylation or SNPs) by requiring a minimum matching sequence length to assure the amplicon assignment. We frequency of mismatches to the predicted converted and unconverted amplicon sequence is depicted in Figure S2. It is evident, that the 95% criterion used typically in SNP discovery experiments is suitable for the converted amplicon reads as well. Using this filter criterion, a further 30–40% of reads were eliminated. Ambiguous reads mapping to different regions of the target sequence were filtered out, too. Primer sequences were removed before methylation scoring. Cytosine methylation in the mapped reads was assessed as the ratio of C to T in the aligned sequences. An overview of the raw sequence reads and the proportion used for final analysis is provided in Table 1. The software and example datasets are available for anonymous download at (http://gengastro.1med.uni-kiel.de/suppl/methyl454/).

**Table 1:** Overview of the samples and reads from the libraries.

Sample	Tissue	bisulfite	reads	Total sequence (bp)	Mapped reads	Mapped bp w/o primers
CRC-NT	colon	YES	302,893	57,977,571	163,971	30,388,486
CRC-TU	colon	YES	193,150	39,029,236	119,666	22,368,077
FL-R1	lymphatic	YES	857,952	186,092,051	681,836	133,755,191
FL-R2	lymphatic	YES	843,643	165,164,339	580,212	105,088,308
CRC-NT	colon	NO	152,873	61,380,483	127,151	48,206,498
CRC-TU	colon	NO	183,423	73,311,754	153,450	57,586,858
FL-R1	lymphatic	NO	167,205	66,481,618	140,562	52,358,040
FL-R2	lymphatic	NO	323,993	109,867,968	223,888	76,258,994

The table gives an overview of the samples and reads obtained from the converted (marked as bisulfite “YES”) and unconverted libraries (marked as bisulfite “NO”). The raw number of sequence reads, the number of mapped reads per sample and total analysed sequence after deletion of primer sequences are listed.

### Analysis of methylation using bisulfite pyrosequencing

Analysis of methylation was performed via bisulfite pyrosequencing as described previously [8], [39]. Primers were designed using the PyroMark Assay Design Software (Version 2.0). The amplification reaction was performed using the PyroMark PCR Kit (Qiagen, Hilden, Germany) according to the manufactures instructions.

### Hepitypes and tree analysis

For each locus of interest only continuous sequence reads covering the region of interest were considered. Only hepitypes with a frequency above 1% frequency in the observed reads for a given sample were considered. Phylogenetic trees were fitted using unrooted maximum-parsimony methods as implemented in Phylip 3.69 (http://evolution.gs.washington.edu/phylip.html) with default parameters. Reproducibility of the phylogenetic trees was assessed using 1000 bootstrap samples. To investigate the hypothesis that single unmethylated sites occur on the background of all-C hepitypesat the same frequency as single methylated sites do on the background of all-unmethylated hepitypes, a 2×2 count table of deviating sites was constructed. To this end, counts of methylated/unmethylated hepitypes without any deviating site formed the first column, whereas hepitype counts with at least one site deviating from the all-methylated/all-unmethylated background entered the second column. Fisher's exact test was then performed on this 2×2 table.

## Results

### Yield and coverage of target regions

**Figure 2:**
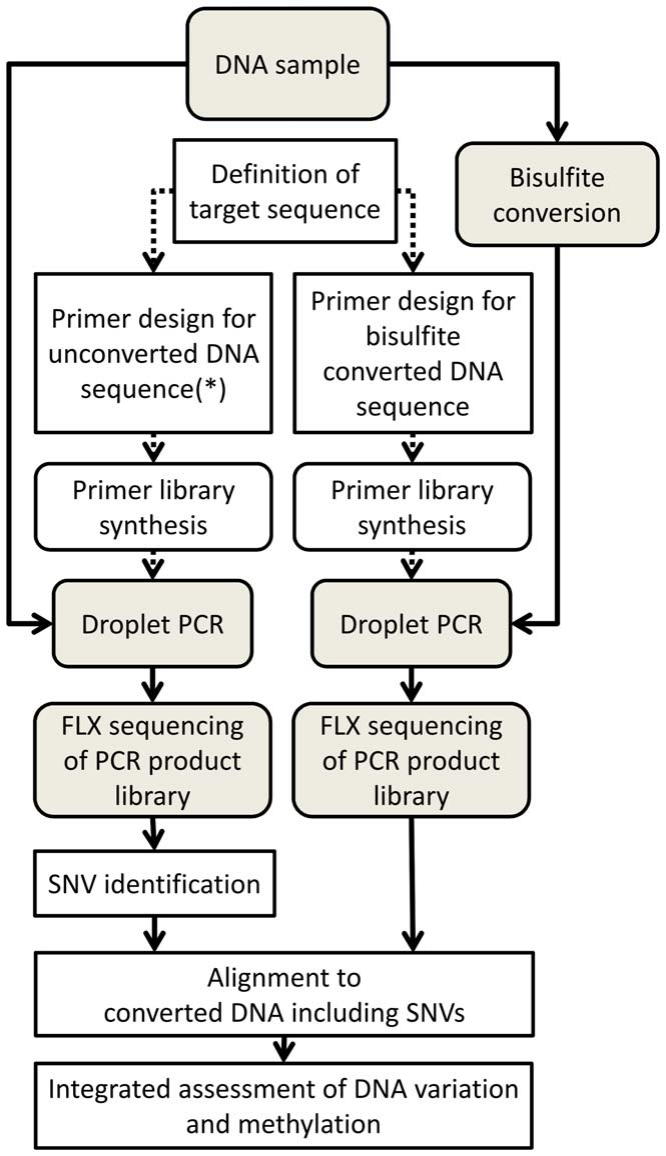
The Figure provides a flow chart of the pipeline for integrated analysis of DNA variation and methylation analysis as described in this manuscript. The standard design pipeline for unconverted DNA [32] is marked with an asterisk (*).

We established a pipeline allowing a simultaneous, targeted assessment of DNA sequence variation and methylation. This pipeline is described graphically in Figure 2. For the predetermined genomic regions of interest, primers for microdroplet amplification of unconverted and BS treated DNA are generated, the primers libraries are synthetized and PCR conditions optimized for maximal product yield. The removal of SNP sites in the primer sequences did not prove to be a critical restriction, than hindered successful primer design in the target regions chosen. The results of this optimization of PCR amplification conditions are depicted in Figure 1 for the 994 primer pairs covering 34083 bases of genomic sequence excluding primer sequences. 946 primer pairs were designed using the automated pipeline for the BS converted DNA. These primers designed redundantly, so as to provide a target coverage of 8-fold over the target sequence. As an additional control, 48 previously established and manually designed primer pairs for CpG islands and a control plasmid were included in the primer library [8]. The theoretical distribution of PCR products is depicted in Panel A of Figure 1. After performing droplet PCR on BS treated DNA as described in the Methods section, the size distribution of amplicons resembled the calculated distribution which is a first indication of a successful design (Figure 1, Panel B). After microdroplet PCR and FLX sequencing of the resulting PCR product library, the sequence reads were aligned to the converted genome as described in the Methods. Using the presence of at least one read of successfully aligned sequence as a criterion, the design success of the automated pipeline averaged at 95% (median 100%). Less than 0.02% of this sequence was covered by less than 20 aligned reads – the corresponding CpG sites are marked in italics in Table S3. Out of the 993 primer pairs targeting the converted human genome in the library, all primers yielded a product, although some sequences cannot be uniquely assigned to one primer pair because of the highly redundant design. Thus, regions not covered in the automated pipeline are due to the failure to design primers for this proportion of the target sequence, rather than experimental lack of amplicons for the designed primers. The one control primer pair for plasmid DNA did not yield a product in any sample.

**Figure 3:**
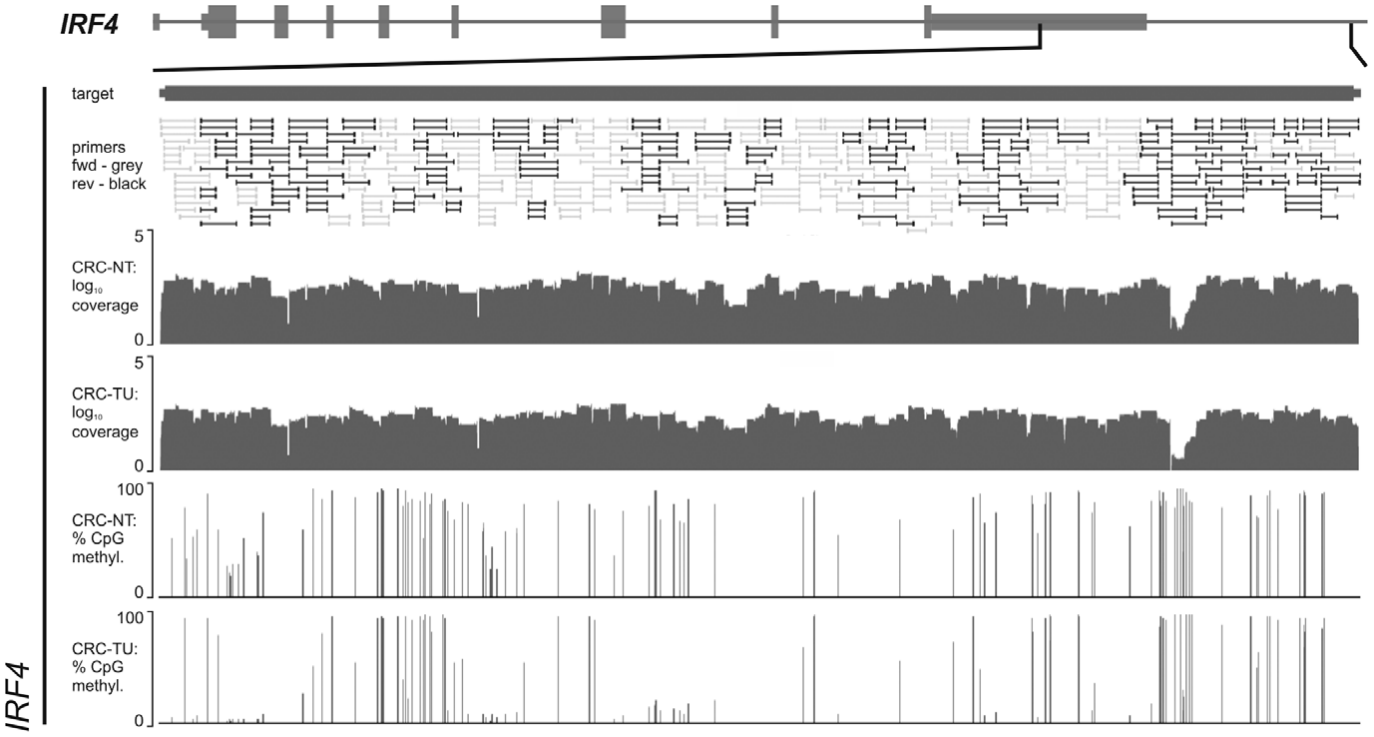
The Figure gives an overview of the amplicon and primer positions of the design, coverage and CpG methylation for the hepityping region *IRF4* (panel B, chromosome 6 from 352,937–366,151 bp). The location relative to the gene model, the primer positions, the log10 coverage and the percentage of methylation for the two colorectal cancer samples are plotted as a function of the genomic coordinates.

A graphical account of the designed primers for the one of the two largest regions with the lower coverage (*IRF4*) is given in Figure 3. Details of the regions and the design and sequencing success of are provided in Tables S1 and 1. The average coverage of the non-converted library was 99.7% (median 100%).

### Technical reproducibility of the levels of DNA methylation

**Figure 4:**
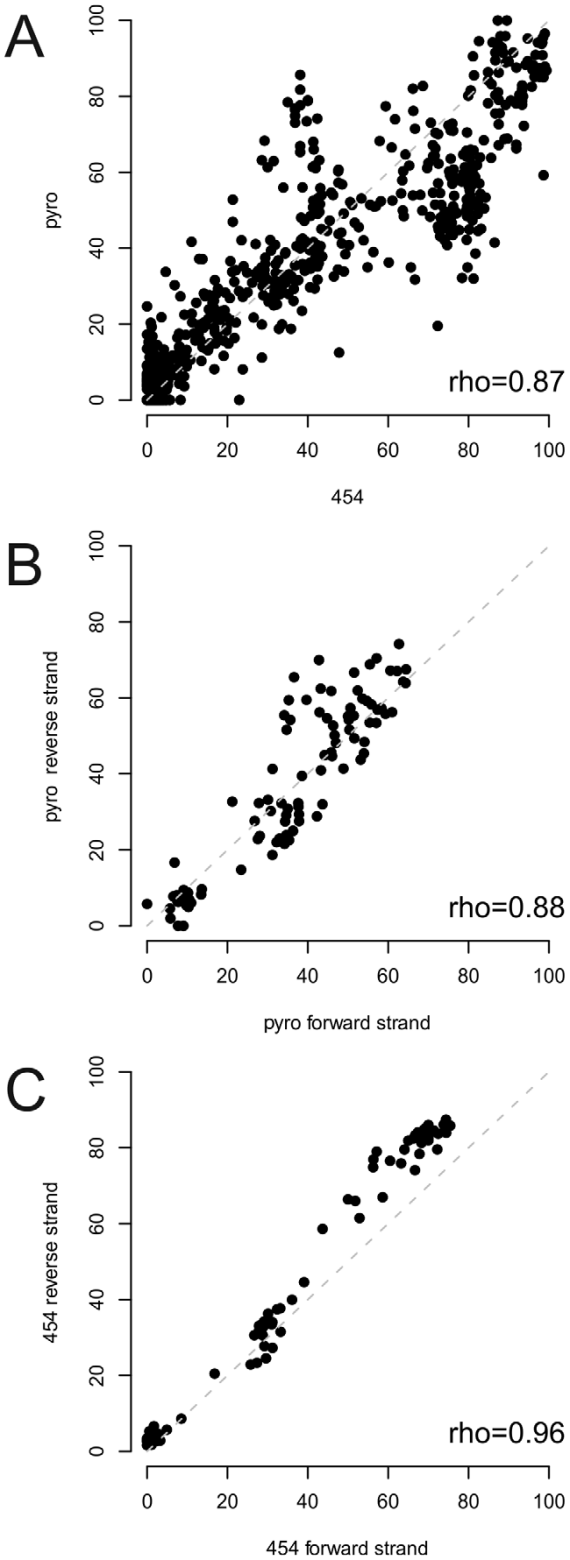
Panel A depicts the correlation of the degree of methylation as measured by pyrosequencing (y-axis, denoted “pyro”) and by sequencing of the droplet-PCR products (x-axis, denoted “454”) for all 248 CpG loci studied with both methods in the panel for all four samples. The Spearman rank correlation coefficient is denoted in the Figure as “rho”. Panels B and C provide a pairwise correlation plot and analysis of the degree of methylation for the forward and reverse strand for 25 CpG sites in the *IGF2* gene.

For 248 CpG sites from 16 loci both BS sequencing data using the author's pipeline and BS pyrosequencing data using conventionally designed control assays (Table S5) were available for comparison. Overall, the degree of methylation as determined by the droplet BS PCR pipeline and by BS pyrosequencing was very similar (Figure 4, Panel A), yielding an overall pairwise Spearman correlation coefficient of 0.87. For the *IGF2* locus (see Table S1 for coordinates), methylation information from the forward and reverse strand of all CpG sites was available from the PCR product sequencing and pyrosequencing assays. Interestingly, the cross-stand reproducibility of the droplet PCR sequencing pipeline was considerably higher than for the pyrosequencing assays, as reflected by the pairwise correlation coefficients of 0.96 and 0.88, respectively (Figure 4, Panels C and B). These results were obtained using primers designed specifically for the opposite strands of the converted DNA.

### Analysis of DNA methylation and methylation haplotypes (“hepitypes”) in tumors

A total of 2017 differentially methylated CpG sites were analyzed in this study. Table S3 provides the detailed, per site methylation information for all four samples investigated. Sites that were present due to particular SNP alleles could be readily identified using the data from the non-converted library and are annotated in Table S3, too.

The readout of the PCR products yielded an average read length of 204 bases. Owing to these relatively long stretches of continuous DNA sequence, an analysis of phased methylation information was possible. We denote the underlying methylation pattern of these phased sequence reads of converted DNA as “hepitypes” [19], [28]. As exemplary loci to demonstrate the feasibility and potential of this experimental approach, the *SNRPN* (small nuclear ribonucleoprotein polypeptide N), *FGF6* (fibroblast growth factor 6) and *HS3ST2* (heparan sulfate 3-O-sulfotransferase 2) loci were evaluated considering phased methylation covered by complete physical sequence reads. A minimum hepitype frequency of 1% was required for analysis.

### *SNRPN* (small nuclear ribonucleoprotein polypeptide N)

**Figure 5:**
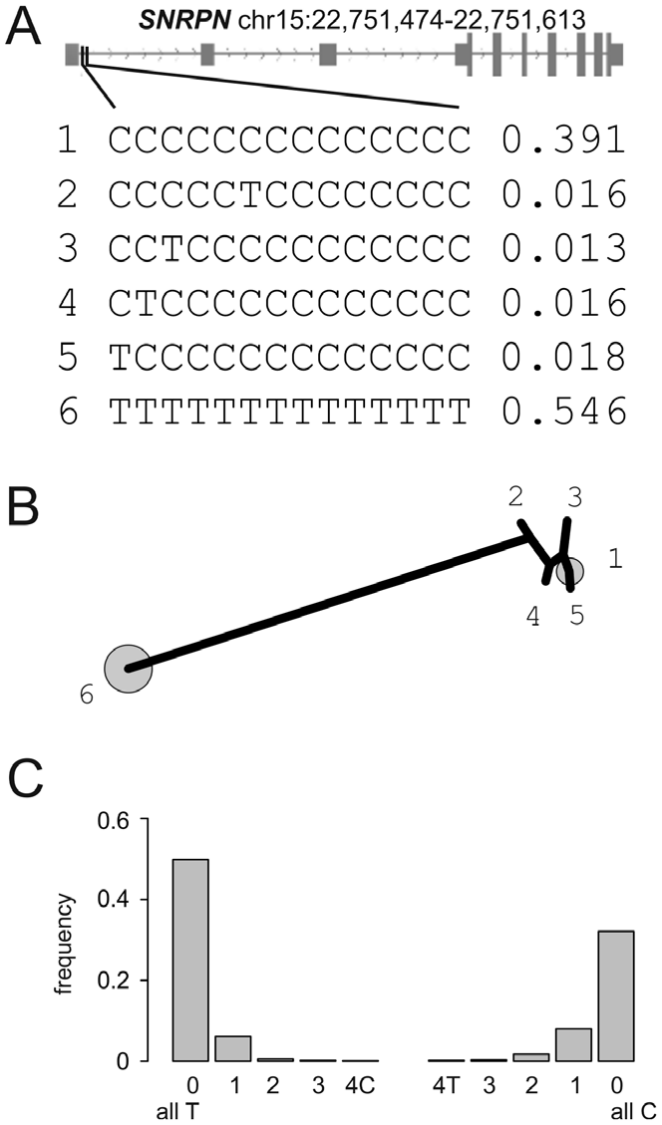
The figure shows the hepitypes of at the CpG island at genomic coordinates on chromosome 15 (22,751,474–22,751,613 bp) at the *SNRPN* (small nuclear ribonucleoprotein polypeptide N) locus for the sample CRC-NT providing an example of an imprinted gene. In panel A, the hepitype frequencies and an overview of the locus structure is given. Panel B shows a maximum-parsimony tree constructed from hepitypes with a minimum frequency of 1%. The numbered hepitypes from panel A are annotated in the tree. The frequent hepitypes are indicated by grey circles sized according to the relative hepitype frequencies. Panel C summarizes the frequency of methylated and non-methylated sites on the background of the two hepitypes (left completely unmethylated – all T, right – completely methylated – all C) defined by the imprinting of the locus. As detailed in the Results section, single unmethylated sites occur more frequently on the background of the all C hepitype, than single methylated sites on the background of the unmethylated hepitype (p<1×10^−15^).

*SNRPN* was investigated as an example of an imprinted gene, i.e. a gene differentially methylated on and expressed from the two parental alleles. The *SNRPN* gene (OMIM 182279) is transcribed exclusively from the paternally inherited chromosome. SNRPN is located within an imprinted gene cluster in chromosome 15 that is associated with Prader-Willi syndrome (PWS; OMIM 176270) and Angelman syndrome (OMIM 105830) [40]. PWS arises from loss of gene function in this region, expressed exclusively from the paternal chromosome; this suggests that SNRPN may play a role in its etiology. Theoretically, given the equal distribution of both parental alleles in a healthy tissue sample, one would expect 50% of all alleles in such a sample to be methylated and 50% to be unmethylated. As shown in Figure 5, the completely unmethylated hepitype and completely methylated locus comprised 55% and 39% of all sequence reads in the studied normal colonic sample (CRC-NT), respectively. Data for all three other samples are almost identical (data not shown). Whereas a dichotomy of hepitypes, reflected in the parsimony tree in Panel B, is expected under the differential methylation of both alleles, the higher diversity of the methylated rather than the unmethylated hepitype caused by incomplete methylation of the supposedly methylated allele is intriguing. Upon formal analysis of the hepitype data, single unmethylated sites on the background of the completely methylated hepitype are more common when contrasted with single methylated sites on the background of the completely unmethylated hepitype not only in the normal sample (CRC-NT, p<1.0×10^−15^) but also in all the three tumor samples (all p-values<1.0×10^−15^). This observation is graphically depicted in the histogram plotting the frequency of additional methylated or demethylated sites on the respective hepitype background in Panel C of Figure 5. Overall, these findings of the in-depth BS sequencing analysis of this imprinted locus using our novel pipeline might suggest that the active DNA-methyl transferase (DNMT) driven process of DNA methylation maintenance might be prone to mistakes in somatic cells. Similar data were obtained for a second imprinted locus (*RB1*, full data in Table S3) [41].

### *FGF6* (fibroblast growth factor 6)

**Figure 6:**
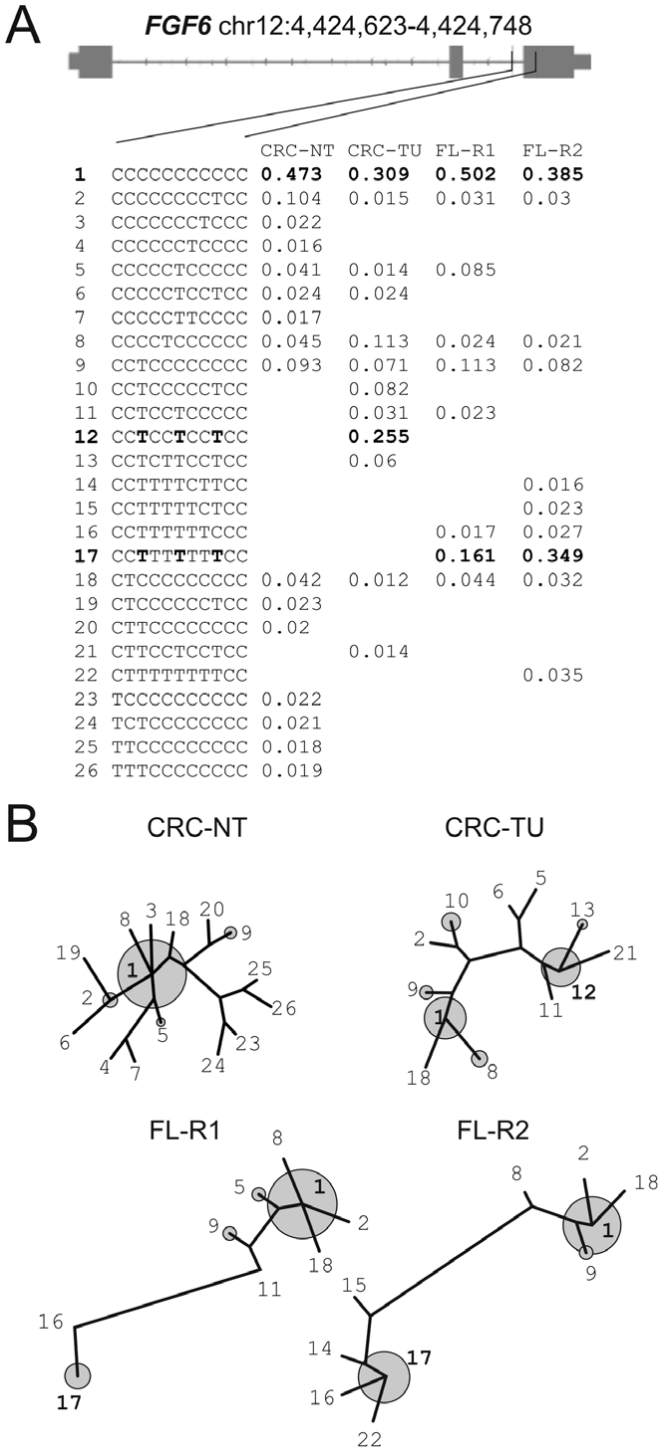
Hepitype diversity at the *FGF6* (fibroblast growth factor 6) locus on chromosome 12 (4,424,623–4,424,748 bp) is characterized by progressive demethylation in tumors. In panel A, the hepitype frequencies in the four samples and an overview of the locus structure is given. Panel B shows maximum-parsimony trees constructed from hepitypes with a minimum frequency of 1%. The numbered hepitypes from panel A are annotated in the trees. The frequent hepitypes are indicated by grey circles sized according to the relative frequencies. Trees have been constructed separately and branch lengths are thus not to scale. It is evident, that both in comparison between normal tissue and tumor in the colon cancer sample (CRC-NT, CRC-TU) and in comparison between the follicular lymphoma and it's recurrence (FL-R1, FL-R2), the locus is characterized by progressive demethylation. The consistently demethylated sites between the colon cancer and lymphoma between hepitypes 12 and 17 are marked in bold print.

Hepitypes located on the CpG island in the *FGF6* gene and the corresponding maximum-parsimony trees are depicted in Figure 6. While the island is largely methylated in the normal colonic tissue specimen (CRC-NT), the central CpGs of this locus are demethylated in the matching colorectal tumor. The respective hepitype 12 is only observed in the CRC-TU samples (25.5% frequency). The follicular lymphoma shows a different demethylation pattern (hepitype 17), which nevertheless involves the same region of the CpG island. Interestingly, the frequency of the partially demethylated hepitype and the degree of demethylation increases in the follicular lymphoma with disease progression in comparison of FL-R1 to FL-R2. Three sites overlap between the predominant demethylated hepitypes for the follicular lymphoma and colon cancer sample (hepitypes 12 and 17 in Figure 4, sites marked in bold print). The common telomeric demethylated position (at 4,424,667 bp, left most T in bold print in hepitypes 12 and 17) overlaps with a TAF1 site. TAF1, a protein found at the start of transcribed genes, is a general transcription factor that is a key part of the pre-initiation complex found on the promoter and might, thus, be involved in deregulation of the FGF6 growth factor.

### *HS3ST2* (heparan sulfate 3-O-sulfotransferase 2)

**Figure 7:**
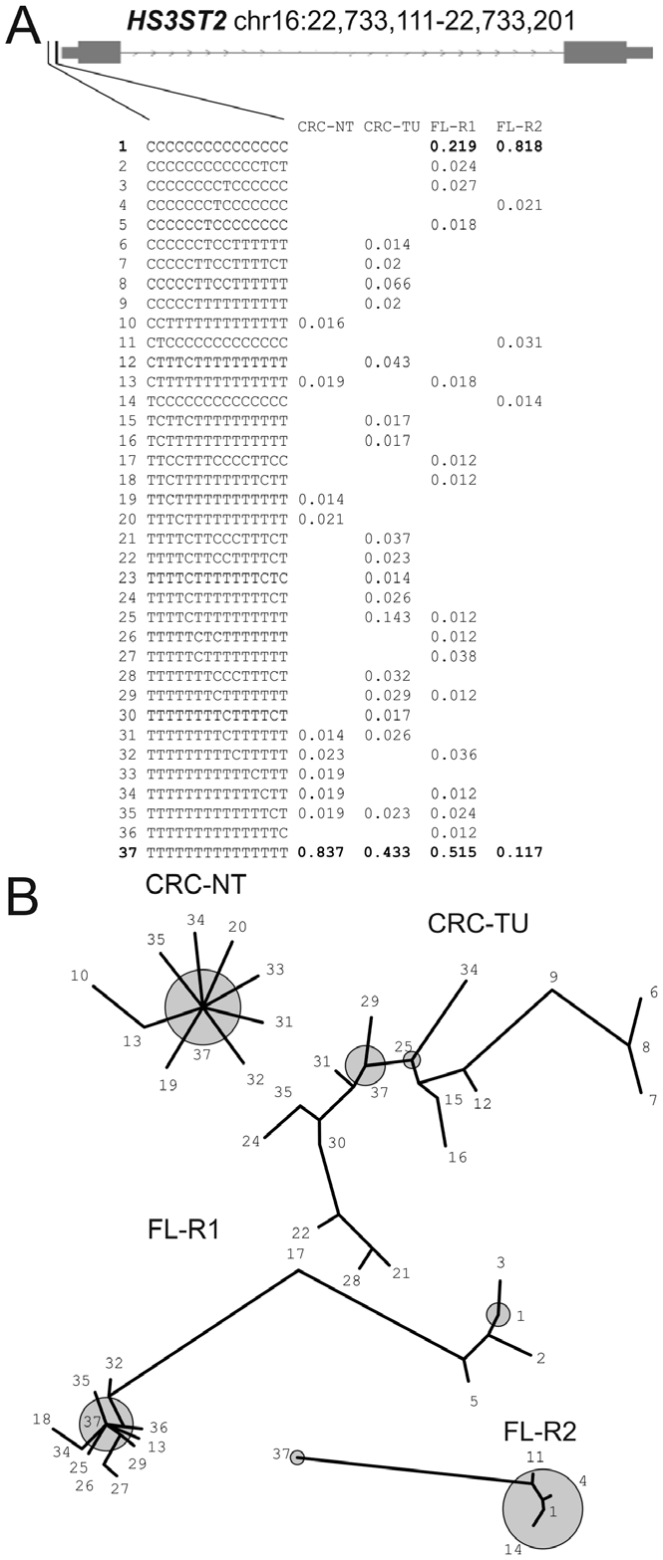
Hepitype diversity at the *HS3ST2* (heparan sulfate 3-O-sulfotransferase 2) locus on chromosome 16 (22,733,111–22,733,201 bp) is characterized by increasing methylation in tumor samples. In panel A, the hepitype frequencies in the four samples and an overview of the locus structure is given. Panel B shows maximum-parsimony trees constructed from hepitypes with a minimum frequency of 1%. The numbered hepitypes from panel A are annotated in the trees. The frequent hepitypes are indicated by grey circles sized according to the relative frequencies. Trees have been constructed separately and branch lengths are thus not to scale. It is evident, that both in comparison between normal tissue and tumor in the colon cancer sample (CRC-NT, CRC-TU) and in comparison between the follicular lymphoma and it's recurrence (FL-R1, FL-R2), the locus is characterized by progressive methylation yielding for instance an 82% frequency of the completely methylated hepitype in the recurrent lymphoma (FL-R2).

The hepitype structure of *HS3ST2* as depicted in Figure 7 shall serve as an example of a locus that is being progressively methylated in the tumor samples studied. As illustrated by the maximum-parsimony trees, hepitype 37 (completely unmethylated) is the dominant hepitype in the normal colon tissue (83.7% abundance), with few randomly distributed single methylated CpGs (e.g. hepitypes 31 to 35). In the tumor sample, a significant proportion of sites are methylated. This process is most clearly visible in the progression of the follicular lymphoma from FLR1- to FL-R2. In the first biopsy, hepitype 37 (completely demethylated) is still the dominant hepitype, whereas in the second recurrence, hepitype 1 (completely methylated) has increased in frequency from 21.9% to 81.8%, corresponding to a “shift” from left to right in the maximum-parsimony trees (Figure 7, panel B).

## Discussion

We report the adoption of the recently described mircodroplet PCR [32] to bisulfite treated target DNA and established a pipeline for the simultaneous assessment of DNA sequence analysis and methylation. We show that successful assessment of methylation status can be achieved for a wide range of target sequences, with an average of 95% of target sequence (median 100%). In principle, this approach is similar to a previously reported DNA methylation profiling experiment on chromosomes 6, 20 and 22, that also used PCR products from bisulfite converted DNA and applied Sanger sequencing to the resulting products [29]. Here, we utilize the advantages of microdroplet PCR and second-generation sequencing technology. We used approximately 1000 primer pairs to cover some 34 kb of genomic sequence – *i.e.* approximately one primer every 34 bp with a 100% success rate (see Results). The efficiency and scalability of the approach depends on the structure of the genomic regions of interest. If large numbers of small sites (e.g. well defined CpG islands) are studied, the design and sequencing overhead of primer sequences is greater, whereas extended hepityping regions (such as *SMAD7* and *IRF4* in this study) can be more efficiently covered. For instance, the total sequence spanned by the amplicons including primers was 42455 bp in this study. The pipeline has a considerable scalability potential, because the degree of redundancy may be reduced, the proportion of longer amplicons might be increased and larger numbers of primers (up to 4000 in the current RainDance technical specifications) may be incorporated in the libraries.

In the range of possible methods that have been developed in order to increase throughput and ease of methylation analysis [26], [27], [28], this targeted approach may be useful, because it allows a selective and thus economical use of sequencing resources. Sequencing depth may be adjusted to achieve the necessary precision for methylation and hepitype assessment. The PCR-based approach also facilitates the alignment of the resulting sequence reads, which may lead to ambiguous results in whole-genome experiments due to the decreased complexity of sequencing reads in bisulfite-treated target sequences. The feasibility of measuring differential methylation over large regions, for example, the peak regions of a susceptibility region for a complex cancer such as colon cancer or lymphoma is demonstrated in this report (Figure 3).

We assessed the validity of the methylation readings from our pipeline approach with a set of 18 manually designed pyrosequencing assays analysing 248 CpG sites and found a good concordance yielding a pairwise correlation coefficient of 0.87. Interestingly, the reproducibility of strand readings was higher for the sequencing reads (rho = 0.96) as compared to the degree of methylation as measured by pyrosequencing from both strands (rho = 0.88). Thus, the comparison to BS pyrosequencing as a gold standard may be overly conservative towards the presented pipeline approach.

We deliberately chose a sequencing technology that delivers relatively long continuous stretches of sequence information, i.e. the Roche FLX system. The average read length in this experiment was 204 bases, allowing us to obtain phased methylation information and the construction of “hepitypes”. These hepitypes provide the potential to study phylogenetic traces of somatic evolution in cancer, such as recently demonstrated for SNP variation by Campbell et. al. [23]. The potential of our pipeline in the investigation of somatic methylation evolution is demonstrated here for three exemplary loci: *SNRPN* (small nuclear ribonucleoprotein polypeptide N), an example of an imprinted gene; *FGF6* (fibroblast growth factor 6) demonstrating a locus with increasing demethylation in cancer; and *HS3ST2* (heparan sulfate 3-O-sulfotransferase 2) exhibiting a pattern of locus methylation in the progress of cancerogenesis. Due to the design as a proof-of-principle experiment, biological conclusions that might be drawn from this limited dataset must be viewed with considerable caution. However, we demonstrate a consistent and statistically significant skew in single base methylation and demethylation on the background of the imprinted hepitypes at the *SNRPN* locus towards incomplete methylation of the imprinted (methylated) hepitype in all samples studied. This may be due to the active nature of the methylation process. The progressive contextual hepitype methylation and demethylation as seen in *FGF6* and *HS3ST2* may provide a paradigm for further studies, especially if different tumor entities with the same direction of overall methylation change but a different hepitype pattern are analyzed. This approach may allow an easier pinpointing of functionally relevant sites of differential methylation as suggested in the hepitype patterns at the *FGF6* locus (Figure 6).

In summary, we present a novel approach for the targeted assessment of differential methylation using microdroplet PCR and second generation sequencing and show its utility for the analysis of hepitype phylogeny in the somatic evolution in cancer.

## Supporting Information

Figure S1 **Verification of successful bisulfite conversion: The figure shows the pyrosequencing trace of the sample CRC-TU at the SMAD7 locus (chromosome 18, position 46,448,939–46,448,969).** The same converted sample, which was used for the sequencing and an analysis pipeline presented in the manuscript, was analysed with bisulfite pyrosencing. For the internal bisulfite control position a “c” is injected before and after the converted “t“. No light signals were obtained for these positions demonstrating complete and successful bisulfite conversion.
(TIF)

Figure S2 **The figure provides the rationale for choosing 95% sequence identity as a criterion for the selection of converted amplicon reads in the analysis.** The dashed line shows the frequency of reads with the identity to the predicted amplicon sequence on the x-axis. The 95% criterion that is customary for unconverted SNP discovery experiments thus appears to be suitable for the converted amplicons as shown in the solid lines.
(TIF)

Table S1 **Overview analysed regions.** For some of the smaller regions only manual design using previously designed or only automatic design was attempted. Regions, where the respective design method was not used are indicated by “n/a”. The large hepityping regions were only processed through the automated pipeline. The coordinates are given according to NCBI build 36.1 (UCSC hg18). The empirical coverage by sequencing reads exactly corresponds to the theoretically expected one and is thus not provided separately.
(XLS)

Table S2 **List of all primer pairs and amplicon yield in the experiment: The primer sequences and target genes are provided.** The mean number of aligned finished reads for each amplicon normalized on 100,000 reads over all four libraries is provided in the column “normalized mean” in addition to the raw sequence counts per amplicon and the normalized (on 100,000 reads per library) reads for each of the four samples.
(XLS)

Table S3 **The table provides a list of all methylated sites in the analyzed samples.** Genomic coordinates refer to NCBI build 36.1 (UCSC hg18). CpG sites that are generated through a SNP are marked in underlined italics. Sites covered by less than 20 reads are marked in non-underlined italics. Sites with no reads in the respective sample are indicated with a dash (“-”). Sites without a CpG in a particular sample are marked with “NA”. The percentage of methylation is given for each site.
(XLS)

Table S4 **List of all polymorphic sites (SNVs and InDels) in the individual samples.** The chromosome, chromosomal position, alleles and individual genotypes are provided for all samples.
(XLS)

Table S5 **The table provides an overview of the 18 manually designed pyrosequencing assays for the replication of the measurement of DNA methylation at 17 selected loci.** Note, that for the *IGF2* locus, an assay on the forward and the reverse strand has been designed.
(XLS)
