# Supplementary figures and images for: Pipeline for Large-Scale Microdroplet Bisulfite PCR-Based Sequencing Allows the Tracking of Hepitype Evolution in Tumors
Source: PLoS One. 2011 Jul 5;6(7):e21332. doi: 10.1371/journal.pone.0021332 (PMC3130030; doi:10.1371/journal.pone.0021332)

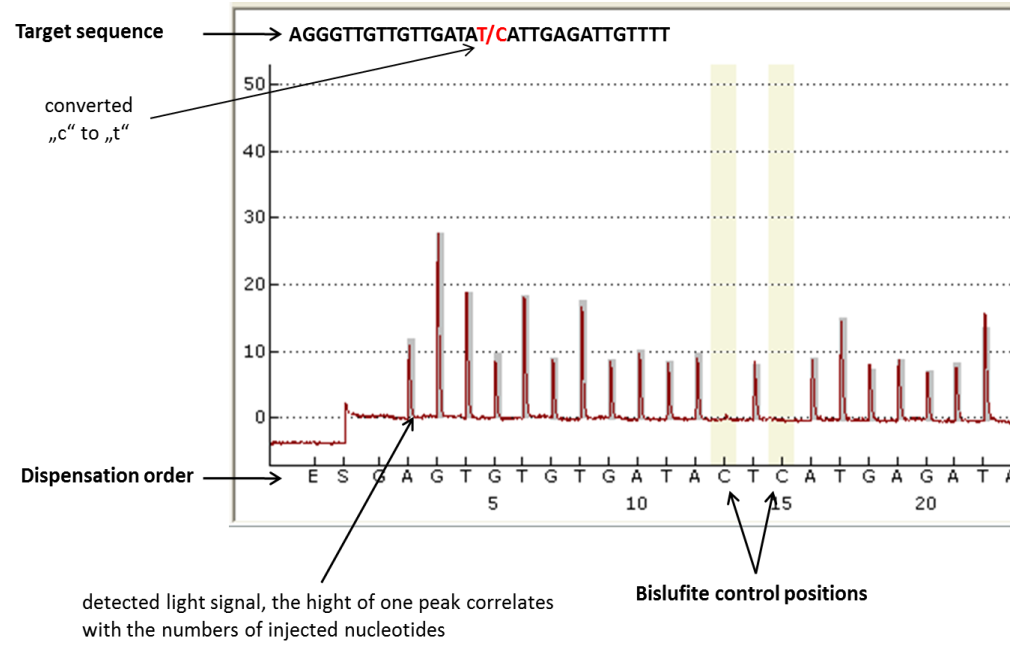

Supplement: Figure S1 — Verification of successful bisulfite conversion: The figure shows the pyrosequencing trace of the sample CRC-TU at the SMAD7 locus (chromosome 18, position 46,448,939–46,448,969). The same converted sample, which was used for the sequencing and an analysis pipeline presented in the manuscript, was analysed with bisulfite pyrosencing. For the internal bisulfite control position a “c” is injected before and after the converted “t“. No light signals were obtained for these positions demonstrating complete and successful bisulfite conversion. (TIF) [file pone.0021332.s001.tif]

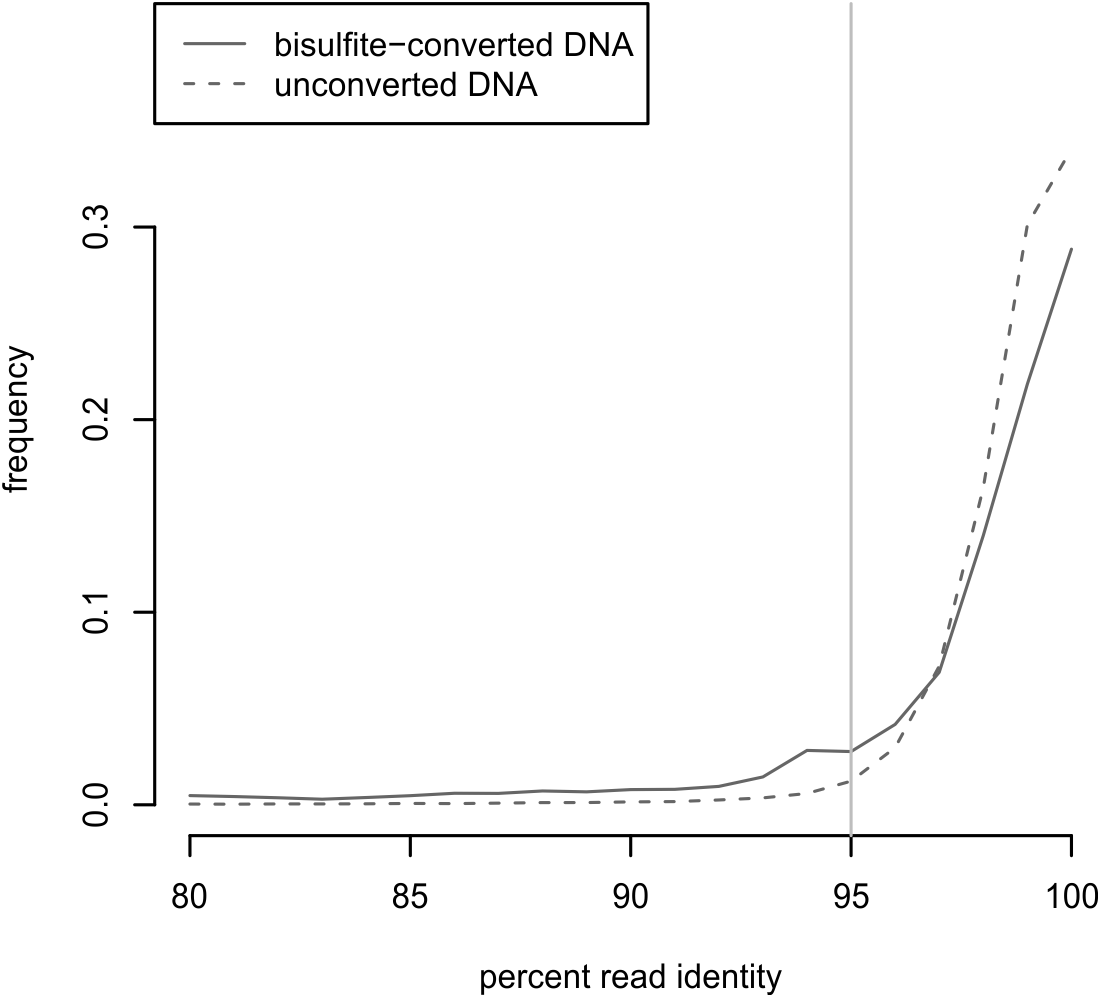

Supplement: Figure S2 — The figure provides the rationale for choosing 95% sequence identity as a criterion for the selection of converted amplicon reads in the analysis. The dashed line shows the frequency of reads with the identity to the predicted amplicon sequence on the x-axis. The 95% criterion that is customary for unconverted SNP discovery experiments thus appears to be suitable for the converted amplicons as shown in the solid lines. (TIF) [file pone.0021332.s002.tif]
